# Senescence Meets Dedifferentiation

**DOI:** 10.3390/plants4030356

**Published:** 2015-06-29

**Authors:** Yemima Givaty Rapp, Vanessa Ransbotyn, Gideon Grafi

**Affiliations:** French Associates Institute for Agriculture and Biotechnology of Drylands, Jacob Blaustein Institutes for Desert Research, Ben-Gurion University of the Negev, Midreshet Ben-Gurion, 84990 Israel; E-Mails: yemimagr@gmail.com (Y.G.R.); vanessaransbotyn@hotmail.com (V.R.)

**Keywords:** senescence, dedifferentiation, chromatin structure, ribosome biogenesis, transposable elements, reversal of senescence

## Abstract

Senescence represents the final stage of leaf development but is often induced prematurely following exposure to biotic and abiotic stresses. Leaf senescence is manifested by color change from green to yellow (due to chlorophyll degradation) or to red (due to *de novo* synthesis of anthocyanins coupled with chlorophyll degradation) and frequently culminates in programmed death of leaves. However, the breakdown of chlorophyll and macromolecules such as proteins and RNAs that occurs during leaf senescence does not necessarily represent a one-way road to death but rather a reversible process whereby senescing leaves can, under certain conditions, re-green and regain their photosynthetic capacity. This phenomenon essentially distinguishes senescence from programmed cell death, leading researchers to hypothesize that changes occurring during senescence might represent a process of trans-differentiation, that is the conversion of one cell type to another. In this review, we highlight attributes common to senescence and dedifferentiation including chromatin structure and activation of transposable elements and provide further support to the notion that senescence is not merely a deterioration process leading to death but rather a unique developmental state resembling dedifferentiation.

## 1. Introduction

Senescence is a well-programmed process accompanied by degradation of macromolecules and remobilization of their constituents into other parts of the plants (e.g., seeds, stems, young leaves) (for reviews: [1,2]). During senescence, many hundreds of genes collectively known as senescence-associated genes (SAGs) are upregulated, including genes for transcription factors, kinases, as well as genes encoding for proteases and RNases [3,4,5,6,7]. Leaf senescence is easily visible due to color changes resulting from degradation of chlorophyll turning leaves yellow. One puzzling phenomenon occurring in senescing leaves of many deciduous trees, particularly in North America and East Asia [8], is the turning of green leaves to red on the onset of senescence, due to light-dependent *de novo* synthesis of anthocyanins coupled with chlorophyll degradation [9,10,11]. This phenomenon supports the proposition that leaf senescence does not necessarily reflect cell deterioration that ultimately leads to death but rather a peculiar developmental state accompanied by *de novo* synthesis of metabolites and other molecules that function in protecting senescent leaves from damaging solar light and insect attack [11].

Leaf senescence may be regulated by endogenous hormonal signals that act to induce or inhibit senescence in a tissue or a developmental stage-dependent manner. Leaf senescence may be controlled via the interplay between the various plant hormones [12,13]. Some defense-related hormones including ethylene, jasmonic acid (JA), abscisic acid (ABA) and salicylic acid (SA) are known to accelerate senescence [13,14,15,16]. Some other hormones including auxin, gibberellic acid (GA) and cytokinins (CKs) may delay senescence [13,17,18,19]. This is well exemplified in transgenic plants overexpressing a bacterial gene encoding for isopentenyl transferase (involved in cytokinin biosynthesis) under the control of a senescence-regulated promoter [20]. These transgenic plants showed high levels of cytokinin and a significant retardation of leaf senescence. Also, photosynthesis was maintained at levels comparable to photosynthesis in young, non-senescing leaves. Similarly, tobacco plants expressing the maize homeobox gene knotted1 driven by a senescence-specific promoter (SAG12) displayed a significant delay in leaf senescence that was accompanied by increased leaf cytokinin content [21]. Cytokinins were also implicated in the formation of “green islands” around infection spots of pathogens [22] as well as following infection with the phytophagous leaf-mining moth *Phyllonorycter blancardella* (Lepidoptera), which results in photosynthetically active green patches in otherwise yellow, senescent leaves [23]. Similar effect on senescence was reported for gibberellic acid (GA). Accordingly, senescence in leaf discs of *Taraxacum officinale*, *Rumex* *crispus*, *R. obtusifolius* and *Tropaeolum majus* was retarded upon application of gibberellic acid (GA) even when degradation of chlorophyll and proteins is halfway complete [18,24,25], suggesting that senescing cells are not yet irreversibly committed to death during first stages of senescence.

## 2. Reversal of Leaf Senescence

Multiple reported data led to the suggestion that senescence is not a terminal process but rather a transitory phase that can be delayed, halted or reversed under certain circumstances. Accordingly, removal of young leaves and the shoot apex or application of cytokinins often led yellow leaves to re-green and regain their capacity for photosynthesis (for reviews: [1,26]). Dark-induced senescence of wheat seedlings can be reversed if re-illuminated after two days but not after four days in the dark, implying that dark-induced senescence is complex and composed of a reversible phase followed by irreversible one [27]. Also, in flax, senescing, yellowing cotyledons were induced to re-green by removal of the growing shoot apex. Ultrastructural examination revealed that re-greening of cotyledons was not derived by *de novo* synthesis or division of chloroplasts but by reassembly of senescing plastids [28]. Similar results were reported in *Nicotiana rustica* in which a senescent leaf was induced to re-green by decapitation and cytokinin treatment [29]. Nitrogen depletion-induced leaf senescence in maize, *Hordeum vulgare* and in *Arabidopsis thaliana* was effectively reversed upon re-supply of nitrogen [30,31]. Indeed, transcriptomic and metabolic profiling of nitrogen depletion-induced leaf senescence in *Arabidopsis* revealed that N-starved plants displayed reduction in PSII efficiency, concomitantly with enhanced expression of the senescent marker gene SAG12 and a notable decline in zeatin riboside; all examined parameters were return to normal level upon re-supply of nitrogen [32]. Thus, reversal of senescence highlights a peculiar developmental junction in the life of the plant cell, which is fundamentally different from programmed cell death (PCD), leading Thomas *et al*. [33] to propose a novel view on senescence. Accordingly, senescence represents a process of transdifferentiation, that is, the conversion of one cell type to another, rather than a deterioration process leading to death.

## 3. What Is Dedifferentiation?

In developmental biology, differentiation is defined as a process whereby cells acquire specific characters that drive their function in a complex organism. Often, differentiation has been viewed as a unidirectional process, namely cells lose their developmental potentialities during maturation leading to “terminal differentiation” (for review: [34]). The concept of dedifferentiation suggests that any differentiated cell retains its developmental capabilities (depending on the integrity of its genome) and can, under specific conditions, return to a more primordial state prior to a change in its fate. Early work on cellular dedifferentiation in amphibians addressed, particularly, morphological changes that occur in cells after limb amputation or when somatic cells are cultured *in vitro* [35,36]. Yet, the definitive proof of dedifferentiation is the capacity of cells to further differentiate into cell types, which are different from the original one [37]. Dedifferentiation in plants has often been referred to as callus cells leading to the common, yet erroneous notion that dedifferentiation and reentry into the cell cycle are alike. Similarly, animal stem cells are commonly defined by their developmental capabilities, rather than by their inherent features leading to the incorrect assumption that reentry into the cell cycle for the purpose of “self-renewal” represents an intrinsic feature of stem cells [38,39]. Apparently, dedifferentiation/stem cell state and reentry into the cell cycle (e.g., self renewal) are two distinct processes. Dedifferentiation characterizes the transition of cells from a given differentiated state into a stem cell-like state that confers pluripotency, a process preceding switch in cell fate including reentry to the cell cycle and even a commitment for cell death. In plants, dedifferentiation is commonly induced by various stress conditions and is well exemplified by protoplasts obtained following treatment of leaf cells with cell wall degrading enzymes [40,41,42,43]. The study of protoplasts revealed some of the inherent features characterizing dedifferentiated cells as well as stem cells both in plants and animals. These include open chromatin conformation, disruption of nucleolar structure and function as well as activation of transposable elements [40,41,42,43,44,45].

**Figure 1:**
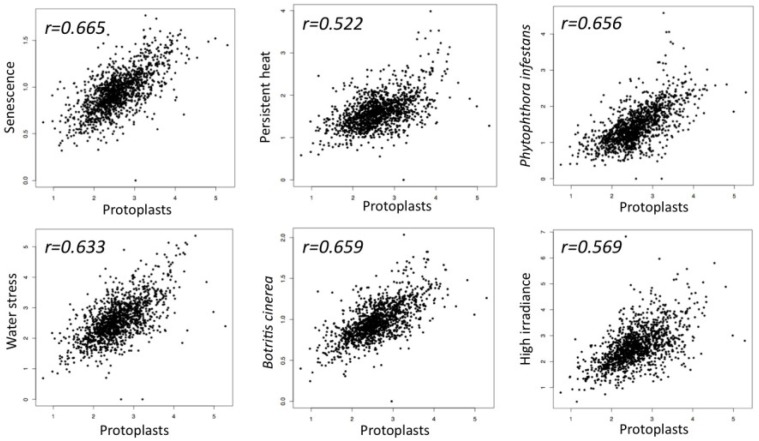
Scatter plots demonstrating similarity in transcription factor expression profiles between dedifferentiating protoplasts and senescing leaves as well as *Arabidopsis* plants responding to various stress conditions including persistent heat, high irradiance, ozone and pathogen infection. Five hundred seventy-six transcription factor encoding genes differentially expressed in dedifferentiating protoplast cells (GEO GSE15515; fold-change ≥ 2, *p* value < 0.05) [42,43] were compared to the expression profiles of *Arabidopsis* senescing leaves (Senescence data were kindly provided by S.H. Wu) [4] as well as to *Arabidopsis* plants responding to high irradiance (GSE 7743), persistent heat (GSE 18666), water stress (GSE 10670), *Phytophthora infestans* (ExpressionSet: 1007966021) and to *Botrytis cinerea* infection (ExpressionSet: 1007967417).

The idea that senescence may represent a transient phase featuring dedifferentiation came from the analysis of the transcriptome profile of dedifferentiating protoplasts and senescing leaves of *Arabidopsis* [4], which revealed unexpected similarities [42]. Particularly, the examination of the expression pattern of transcription factor encoding genes showed [43] that senescing and dedifferentiating cells both display similar expression pattern that also characterizes the expression pattern of TF genes in *Arabidopsis* plants exposed to various stress conditions including pathogen infection, persistent heat, high irradiance and water stress (Figure 1).

Below, we will discuss commonalities between senescence and dedifferentiation with emphasis on chromatin structure, ribosome biogenesis and protein synthesis and TE activation.

## 4. Senescence Meets Dedifferentiation

### 4.1. Chromatin Structure

Presently, it is widely accepted that open chromatin conformation is an inherent feature characterizing the dedifferentiated, stem cell state both in plants and animals [41,44,45]. This feature of stem cells has been described long ago via electron microscope examination of erythropoietic cells during maturation and differentiation. These observations revealed that chromatin appears decondensed in the hematopoietic stem cell and becomes more and more compacted during differentiations [46,47,48]. Some of these observations were supported by the finding that the nucleosome repeat length (NRL) is increased during erythropoiesis in the chick concomitantly with increased concentration of histone H5 [49]—a linker histone necessary for stabilization of higher order chromatin structure [50]. Similar to animal stem cells, electron microscope examination of the shoot apex of the plant *Tradescantia paludosa* showed that a large proportion of the chromatin in the shoot apex cells is organized as diffused euchromatin fibrils [51]. Indeed, cells in the shoot apical meristem of *Arabidopsis thaliana* display a flexible chromatin state demonstrated by overrepresentation of chromatin modifier genes (CMGs) [52]. Accordingly, the analysis of the microarray datasets compiled by Yadav *et al.* [52] revealed that around two third of CMGs in *Arabidopsis* are expressed in the SAM [45]. Dark-induced premature senescence of tobacco leaves displayed a widespread chromatin decondensation and disruption of nucleolar function [42]. Similarly, decondensation of pericentric heterochromatin was reported during leaf senescence in *Arabidopsis* [53,54] and following exposure to long (30 h at 37 °C) heat stress [55]. Indeed, recent data support the hypothesis that plant cells may respond to various environmental cues (commonly inducing premature senescence) by undergoing dedifferentiation characterized by chromatin decondensation and promiscuous expression of transcription factor encoding genes [43,55,56]. Stress-induced dedifferentiation and/or chromatin relaxation is not unique to plant cells and has also been reported in human cells exposed to oxidative stress (paraquat), UV light and hydrogen peroxide [57,58] leading to the hypothesis that mammalian somatic cells may undergo cell dedifferentiation as an adaptation for extreme stress conditions [59,60].

### 4.2. Ribosome Biogenesis and Protein Synthesis

One feature characterizing dedifferentiating cells is the disruption of the nucleolar function. In tobacco, the transition from leaf cells to protoplasts was accompanied by disruption of the nucleolar structure [61]. This might lead to reduction in synthesis of ribosomal subunits and consequently to reduced capacity for protein synthesis followed by acquisition of a quiescent state, a characteristic of stem cells both in plants and animals. Also, plant senescing tissues displayed a decrease in RNA and protein synthesis. Accordingly, senescing cotyledons of soybean (Glycine max) can lose 90% and 80% of their nucleic acids (mostly ribosomal RNAs; no effect on genomic DNA content) and proteins, respectively, before senescence becomes irreversible [62]. Removal of the epicotyl at the time cotyledons turn faint yellow reverses the process of senescence leading to re-greening of cotyledons. Similarly, Makrides and Goldthwaite [63] showed that rRNA and polyribosomes were declined rapidly during maturity and senescence of primary leaves of the bean *Phaseolus vulgaris*, which was accompanied by reduction in protein and chlorophyll content; DNA content remained unchanged even after abscission and withering of the lamina. Skadsen and Cherry [64] reported that ^35^S-methionine incorporation into protein was gradually reduced with the aging of soybean cotyledon but restored within two days after epicotyl removal. In barley and cucumber, leaf senescence was accompanied by loss of polyribosomes and ribosomes and substantial decline in the protein content [65,66]. Thus, the capability of senescing cells to lose large amounts of RNAs and proteins while keeping the integrity of the genome facilitates the acquisition of a quiescent state [67,68] concomitantly with preservation of developmental capabilities.

### 4.3. Activation of Transposable Elements (TEs)

Open chromatin conformation characterizing the dedifferentiation, stem cell state might expose genomic DNA to mutations and genome variation that can be induced by environmental factors such as UV radiation or by activation of TEs, the later may lead to hazardous, neutral or even beneficial effects [69,70]. Acute stress such as tissue culturing and pathogen infection often induces extensive epigenetic modifications and chromatin reorganization that consequently release constraints over TEs resulting in activation and transposition into other chromosomal sites (for review: [71]). In a recent report, Zhu *et al.* [72] have shown that LINE1 retroelement is activated in salamander during limb regeneration. The authors suggested that activation of LINE1 could serve as a marker for dedifferentiation during early stages of limb regeneration. Similarly, TEs were activated in dedifferentiating plant protoplasts. Accordingly, the Ty1-copia retrotransposon was activated in potato (*Solanum tuberosum*) during protoplast isolation [73]. Also, Pouteau *et al.* [74] have shown that the TNT1 retroelement is specifically expressed in leaf-derived protoplasts while in leaf tissue it is silent. The authors suggested that TE activation might provide the molecular basis for some of the somaclonal variation events. In this respect, it is worth mentioning McClintock’s view on cell culturing and on plant stress response in general. McClintock [75] pointed that exposure of cells to acute stress such as pathogen infection and cell culturing might specifically induce genome modification driven by the activation of TEs. Referring to regeneration of plants by tissue culturing McClintock wrote: “It may be safe to state that no two of the callus derived plants are exactly alike, and none is just like the plant that donated the cell or cells for the tissue culture.” Indeed, dedifferentiating protoplasts appeared to be highly potent in activation of TEs inasmuch as 25% of the plants regenerated from tobacco protoplasts displayed newly transposed TNT1 copies compared to less than 3% in plants regenerated from explants culture [76]. Similarly to protoplasting and tissue culturing, various biotic and abiotic stresses including UV light, salt, drought, heat and pathogen attack, which are often inducing premature senescence (for reviews: [2,77]) were reported to trigger TE activation (for reviews: [45,71]).

Our knowledge on TE activation during leaf senescence came from the analysis of gene expression profiles of senescing leaves. For example, transcriptome analysis of early senescing flag leaves of wheat showed upregulation of class I and class II transposable elements [78]. Also, by using the systemic fungal symbiont *Epichloë festucae* and the perennial ryegrass (*Lolium perenne*), Eaton *et al.* [79] showed that the fungal mitogen-activated protein kinase *sakA* is essential for the establishment of the mutualistic interaction. Deletion of *sakA* switches the fungal interaction with the host into a pathogenic one, leading, among other things, to loss of apical dominance and premature senescence. Interestingly, transcriptome analysis of the plant revealed up-regulation of host genes involved in pathogen defense as well as activation of a large number of transposable elements [79]. The analysis of the dataset compiled by Lin and Wu [4] addressing the transcriptome profiles of dark-induced premature senescence of *Arabidopsis* leaves revealed upregulation of several class II transposable elements including members of the hAT (hobo/Ac/Tam3)-like transposases encoded by At1g80020 and *DAYSLEEPER* (At3g42170), the later is an *Arabidopsis* “domesticated” hAT-like transposase found to be essential for plant development [80], En/Spm-like transposon (e.g., At2g40070) and Mutator-like transposase (At2g13970). Notably, *DAYSLEEPER* and At1g80020 were also activated in dedifferentiating protoplasts [42] and in various domains of the shoot apical meristem (SAM) of *Arabidopsis* (based on data assembled by Yadav *et al.* [52]). Interestingly, the analysis of the SAM transcriptome data revealed that a large number of transposable elements are activated in SAM, most of which are class II TEs. Thus the pattern of *DAYSLEEPER* gene expression together with the fact that it is localized at the pericentric region of chromosome 3—a region undergoing decondensation in the course of cell dedifferentiation suggests that expression of *DAYSLEEPER* may be used as a marker for *Arabidopsis* cells acquiring pluripotent state.

Notably, TE activation was reported in animal stem cells as well as in induced pluripotent stem cells (iPSCs) (for review: [81]). Accordingly, human embryonic stem cells (hESCs) were found to overexpress an array of retroelements including the long interspersed nuclear element class 1 (LINE-1 or L1) and the short interspersed nuclear elements (SINEs) Alu; hESCs were also found to support a low level of L1 retrotransposition [82]. Using engineered retrotransposon competent-L1 (RC-L1) Wissing *et al.* [83] have shown that RC-L1 can retrotranspose in iPSCs 10–15-fold higher than in parental fibroblasts. Thus it seems that animal pluripotent cells assume a chromatin environment that allows for transposon transcriptional activation and transposition of otherwise silent TEs.

Multiple studies in animal cells have implicated senescence/aging with activation of TEs. The relationship between TE activation and senescence is complex and appears to be mutual. While senescence was shown to provide the appropriate chromatin environment for activation of TEs, it was also demonstrated that TE activation leads to cellular senescence and death. Accordingly, overexpression of LINE1 ORF2 in MCF7 and HeLa cells resulted in toxicity and reduced cell vitality including cell cycle arrest and apoptosis [84]. This effect was at least partly dependent on the endonuclease activity of the L1 ORF2 protein via the introduction of double strand DNA breaks (DSBs) [85,86]. On the other hand, replicative senescence of normal human diploid fibroblasts was found to be accompanied by de-compaction of chromatin of major retrotransposon classes, including Alu and L1, leading to an increase in their transcription and ultimately transposition [87]. Similarly to plants, animal cells were shown to have the capacity for reversal of senescence. By using *ex-vivo* expansion of human adipose-derived stem cells (hADSCs) to stimulate senescence, it has been demonstrated that senescence of these cells was associated with activation of Alu retrotransposons. However, suppression of Alu transcription reverses the senescent phenotype and enables cells to regain their capacity for proliferation [88]. This suggests that acquisition of the senescence state allows for activation of TEs, which in turn, reinforce the senescent phenotype leading to genome instability and death.

## 5. Concluding Remarks

**Figure 2:**
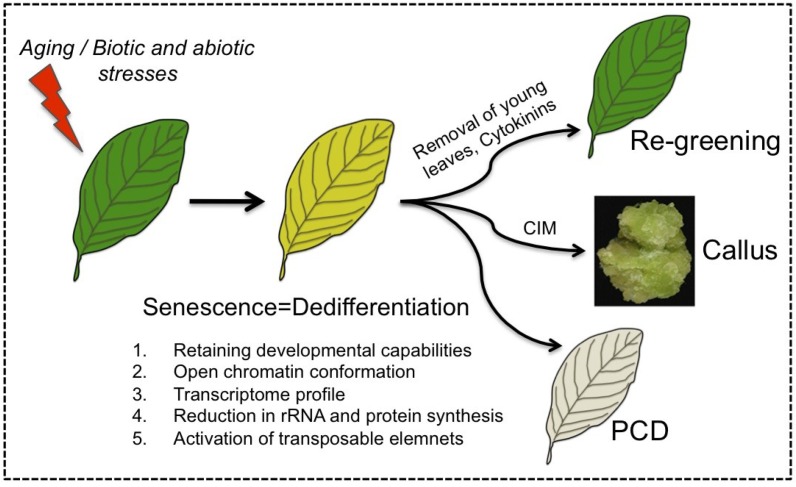
Senescing cells feature dedifferentiation. Leaf senescence is established developmentally as the leaf mature and aged or by biotic and abiotic stresses. Similarly to dedifferentiating cells, senescing cells retain developmental potentialities, acquire open chromatin conformation, and display activation of TEs. Senescing cells can be triggered by various stimuli including removal of young leaves or application of cytokinins to re-green and regain photosynthetic activity, re-enter the cell cycle and form callus upon incubation on callus inducing medium (CIM) [54] or die in an orderly manner (PCD).

The capacity of senescing cells to retain developmental potentialities (e.g., reversal of senescence) distinguishes senescence from PCD and supports the notion that senescence should be viewed as a unique phase that represents a process of dedifferentiation (Figure 2). Accordingly, senescing cells share common features with dedifferentiating protoplasts as well as with cells of the SAM including the retaining of developmental potentialities, open chromatin conformation and activation of transposable elements. Furthermore, various stress conditions such as heat, drought and pathogen infection that induce premature senescence were also found to trigger cells to acquire stem cell features (e.g., open chromatin conformation) [43,54,89]. Since reversal of senescence is a widespread phenomenon found in many dicotyledonous and monocotyledonous plant species, it might have an evolutionary relevance and an adaptive value.

Activation of TEs in the course of cell dedifferentiation indicates that dedifferentiation events should not be viewed simply as a rejuvenation process. Rather, dedifferentiation appears to be a complex process having the potential of recapitulating and accelerating aging processes [90] as well as contributing to somaclonal variation, which is often seen in plants propagated through tissue culture [91]. Accordingly, senescent cells that have regained their previous function or cells that have proliferated from senescing/dedifferentiating cells may have a genome/epigenome, which is different from the one from which they have originated.

Finally, reversal of senescence is assumed to be rare in nature, particularly when referring to deciduous trees where yellow- or red-senescing leaves commonly proceed to death. Thus, why plants had to evolve a mechanism for maintaining developmental capabilities in leaves that are destined for death, and what are the benefits gained by plants from reversal of senescence? Some answers might be related to the sessile lifestyle of plants and their vulnerability to biotic and abiotic stresses that often induce premature senescence.
